# Supplementary figures and images for: Atlas of Fshr expression from novel reporter mice
Source: eLife. 2025 Jan 8;13:RP93413. doi: 10.7554/eLife.93413 (PMC11709436; doi:10.7554/eLife.93413)

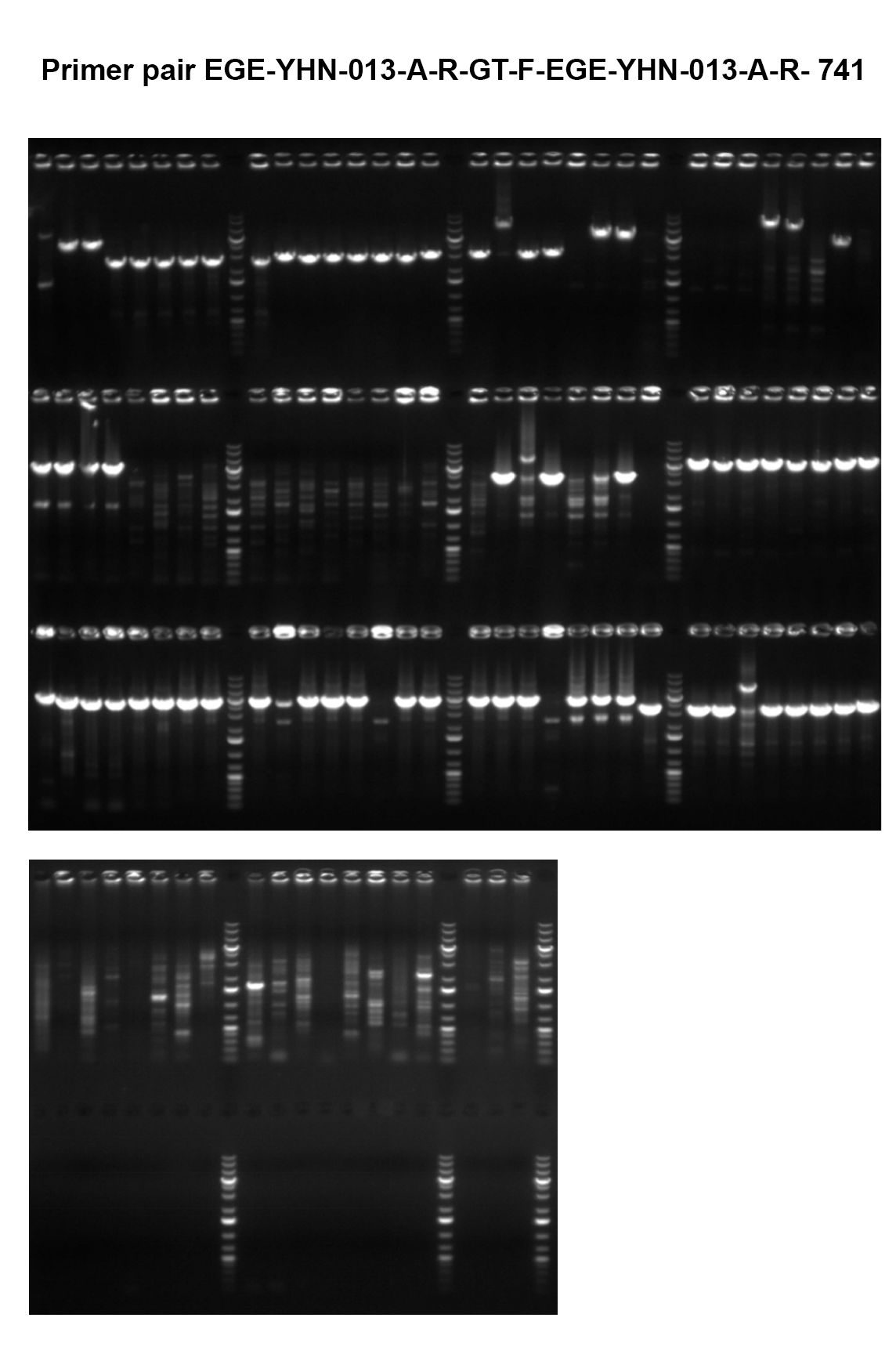

Supplement: Figure 1—source data 1. [file elife-93413-fig1-data1.zip › Figure1B_SourceData/Uncropped Primer pair EGE-YHN-013-A-R-GT-F-EGE-YHN-013-A-R- 741.tif]

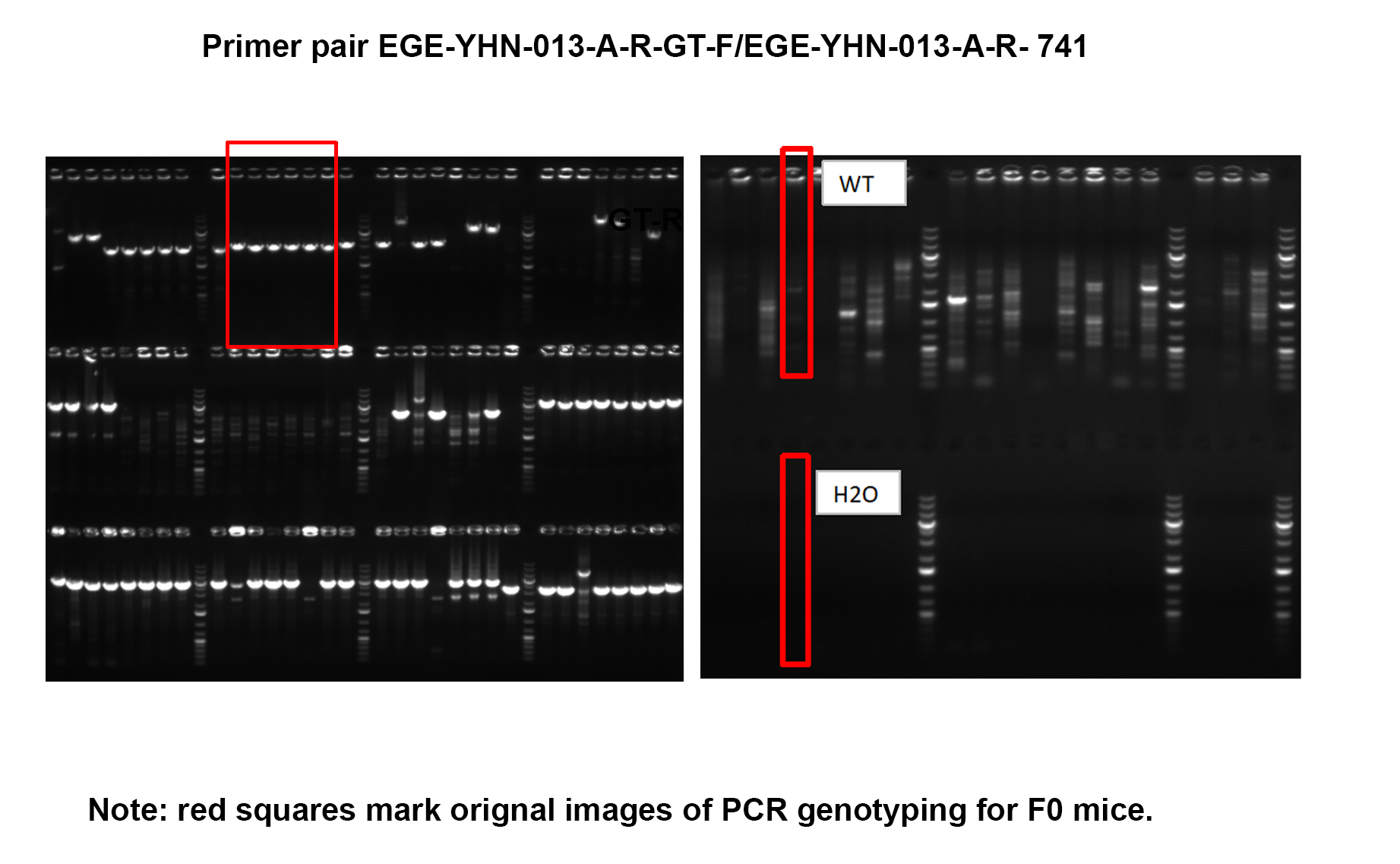

Supplement: Figure 1—source data 1. [file elife-93413-fig1-data1.zip › Figure1B_SourceData/Primer pair EGE-YHN-013-A-R-GT-F-EGE-YHN-013-A-R- 741.tif]

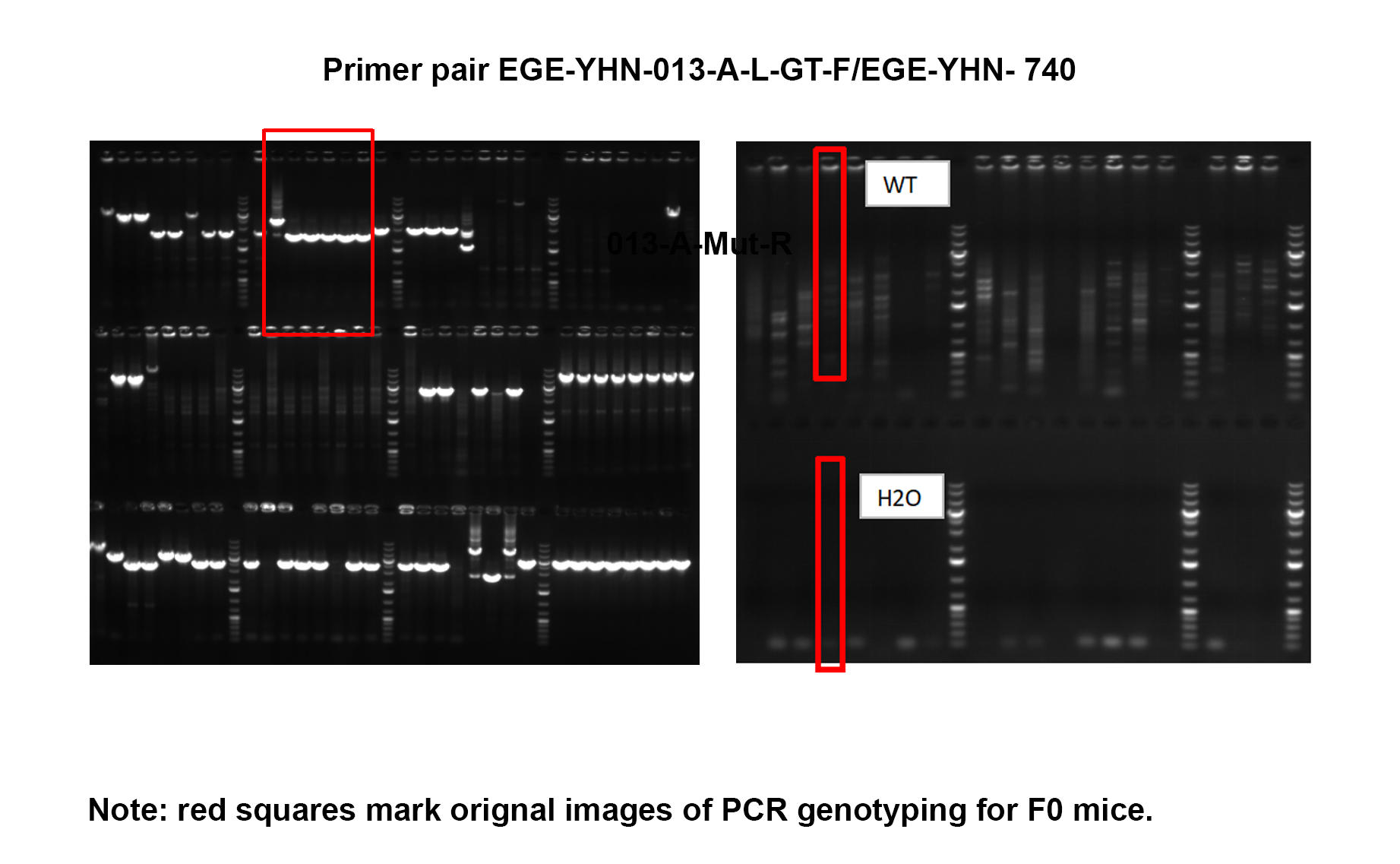

Supplement: Figure 1—source data 1. [file elife-93413-fig1-data1.zip › Figure1B_SourceData/Primer pair EGE-YHN-013-A-L-GT-F-EGE-YHN- 740.tif]

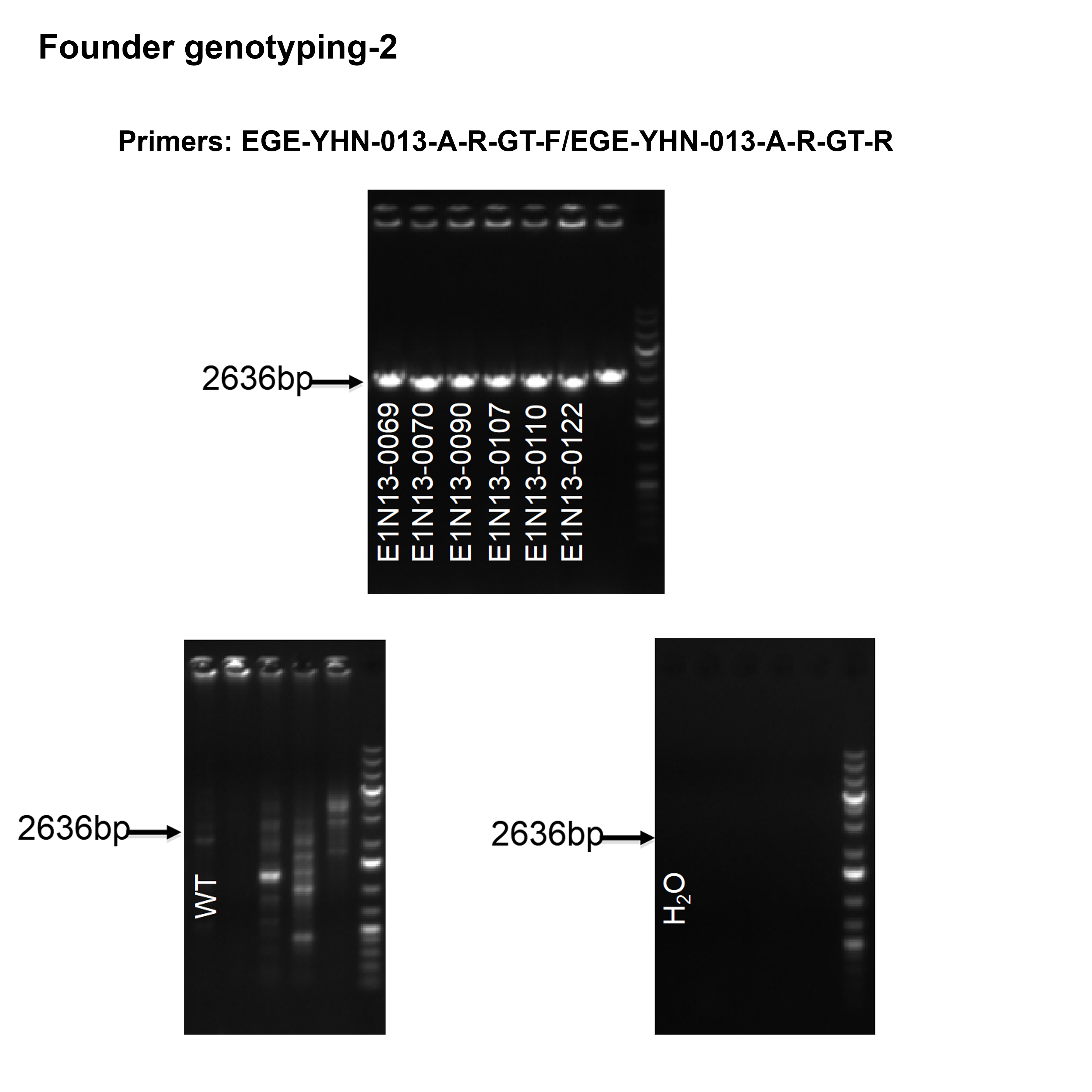

Supplement: Figure 1—source data 1. [file elife-93413-fig1-data1.zip › Figure1B_SourceData/Labelled images for Figure1B-c.tif]

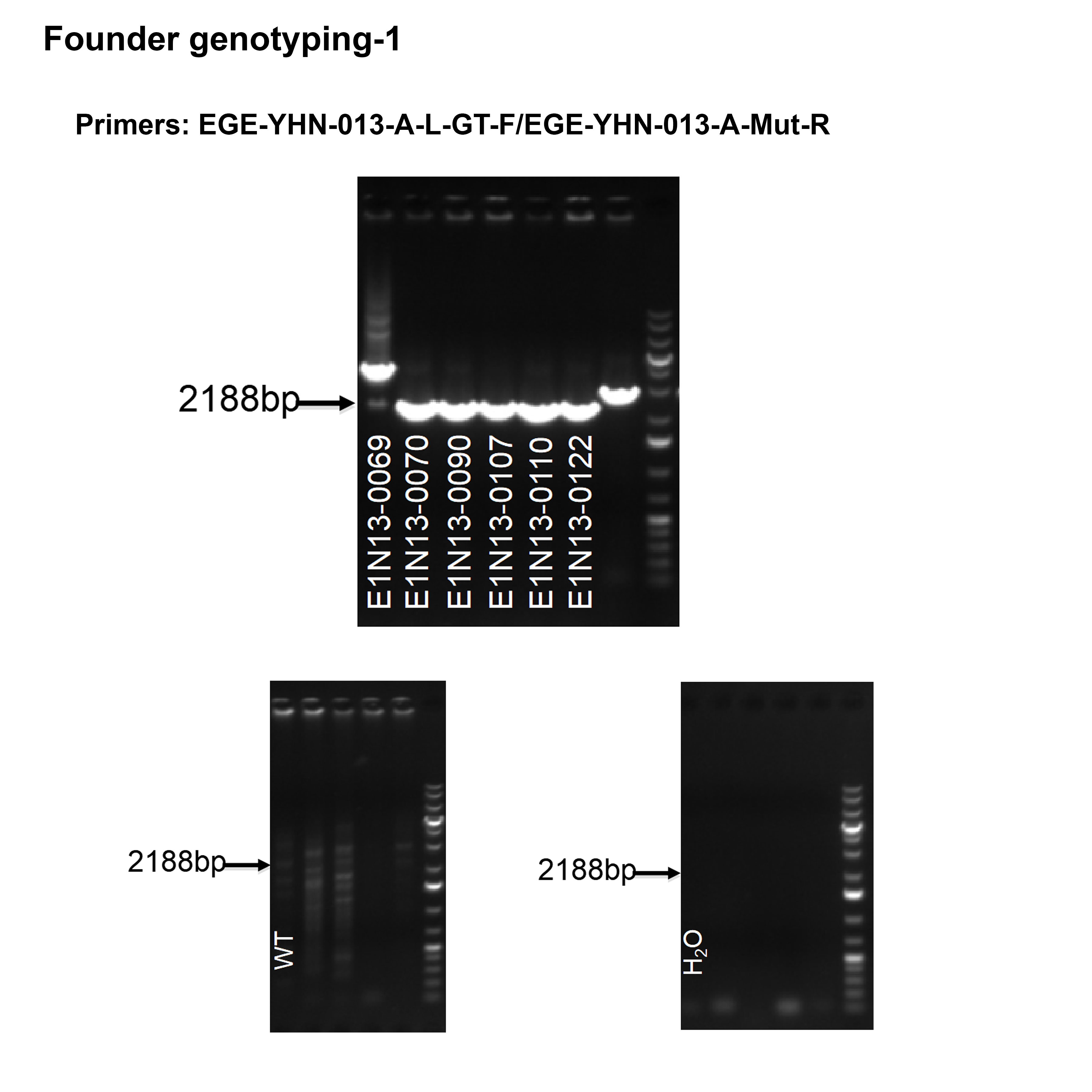

Supplement: Figure 1—source data 1. [file elife-93413-fig1-data1.zip › Figure1B_SourceData/Labelled images for Figure1B-b.tif]

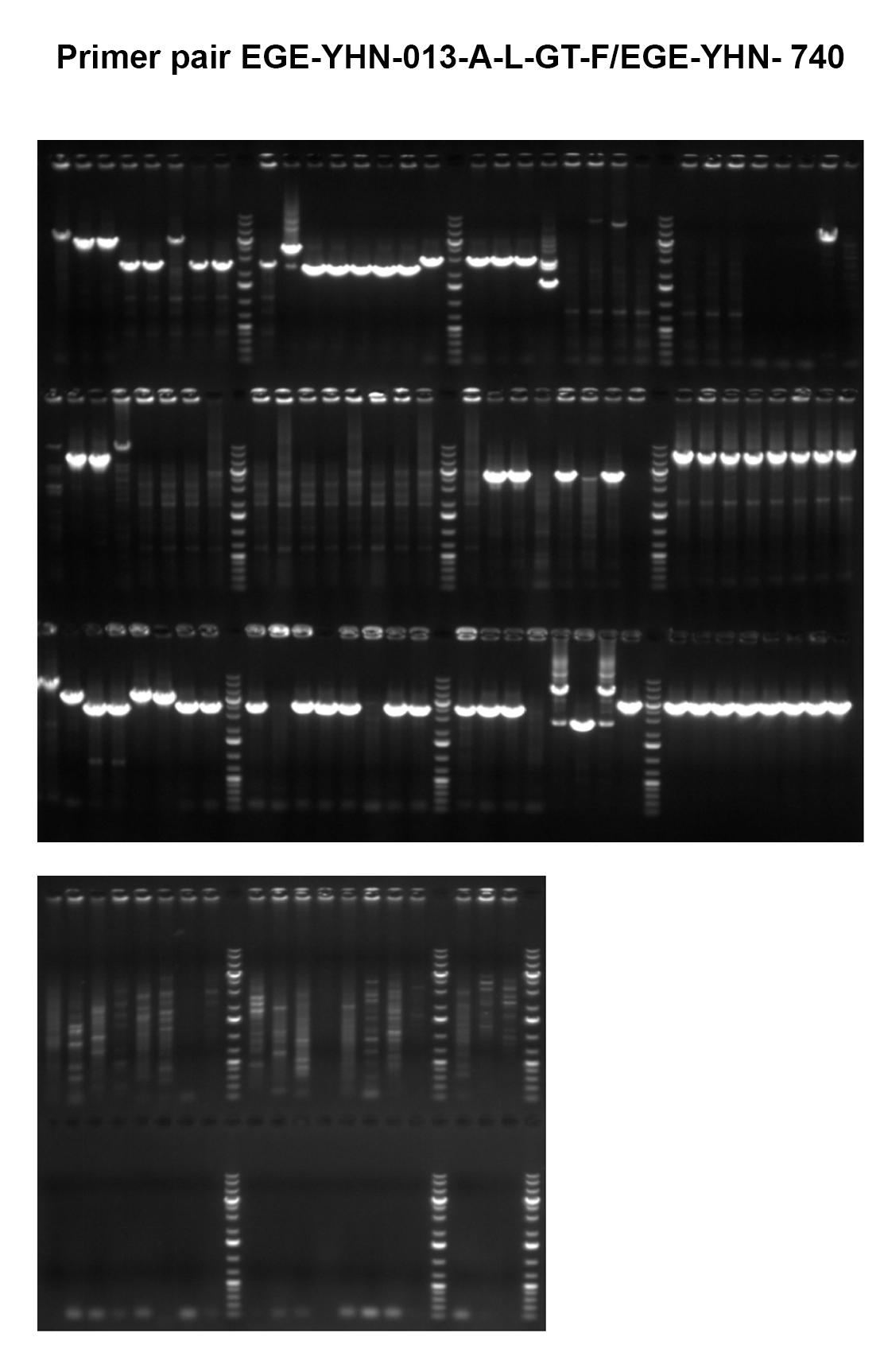

Supplement: Figure 1—source data 1. [file elife-93413-fig1-data1.zip › Figure1B_SourceData/Uncropped Primer pair EGE-YHN-013-A-L-GT-F and EGE-YHN- 740.tif]

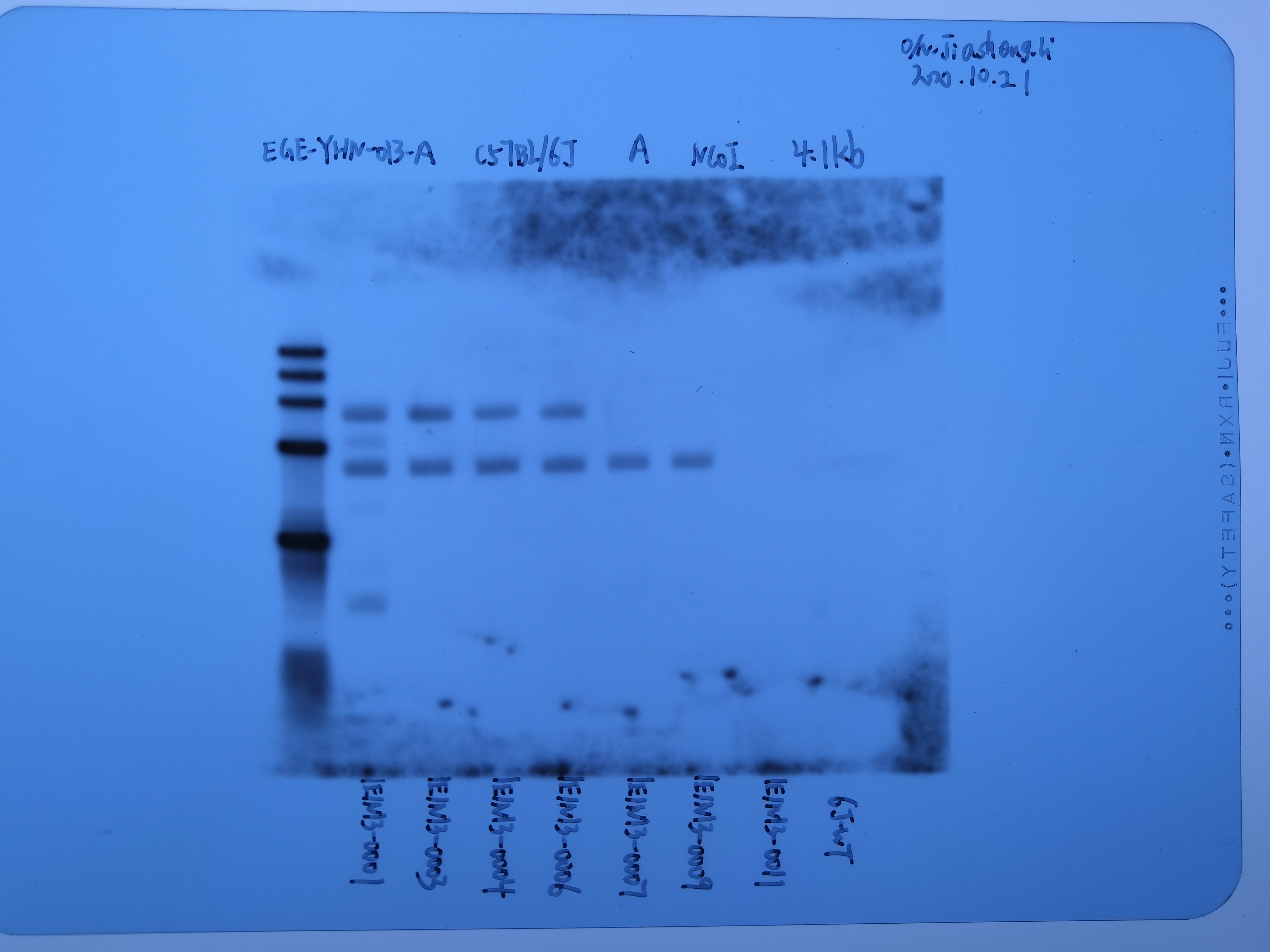

Supplement: Figure 1—source data 2. [file elife-93413-fig1-data2.zip › Figure1C_SourceData/Uncropp original EGE-YHN-013-A A NcoI heter- mice Southern blot original data.tif]

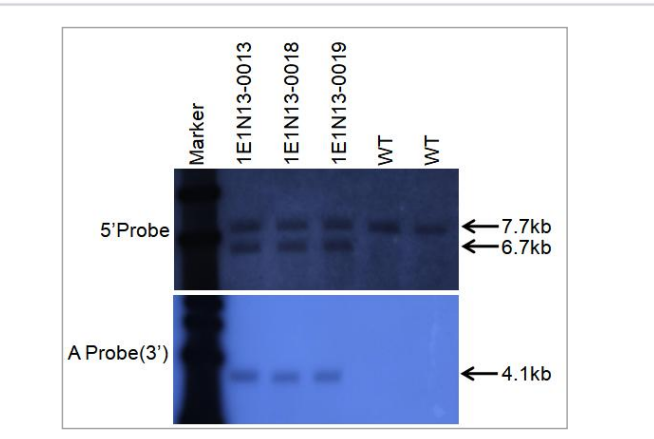

Supplement: Figure 1—source data 2. [file elife-93413-fig1-data2.zip › Figure1C_SourceData/labelled Original images for Figure 1-C-b2.tif]

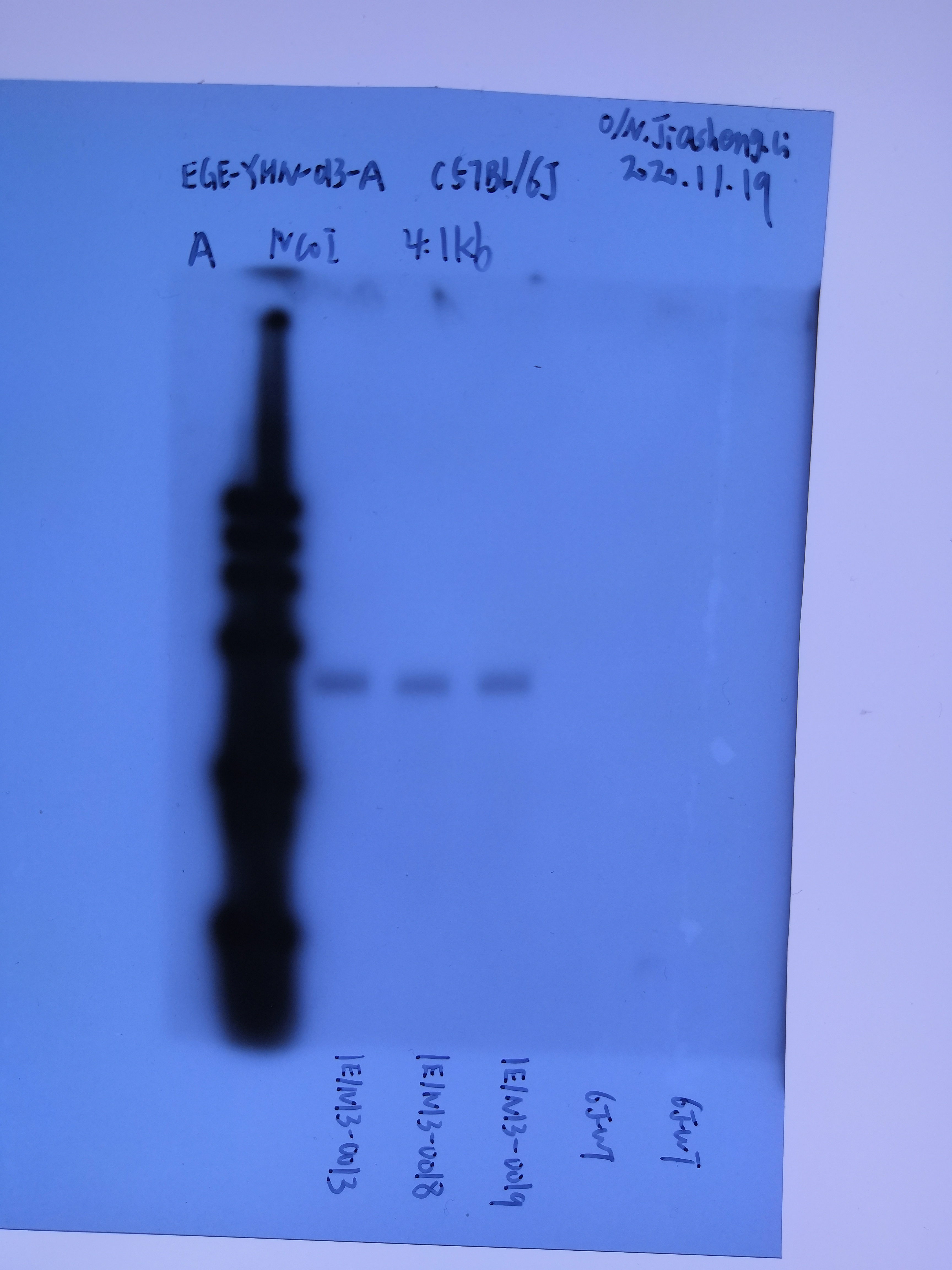

Supplement: Figure 1—source data 2. [file elife-93413-fig1-data2.zip › Figure1C_SourceData/Uncropp original EGE-YHN-013-A A NcoI Heter-mice Southern blot original data.tif]

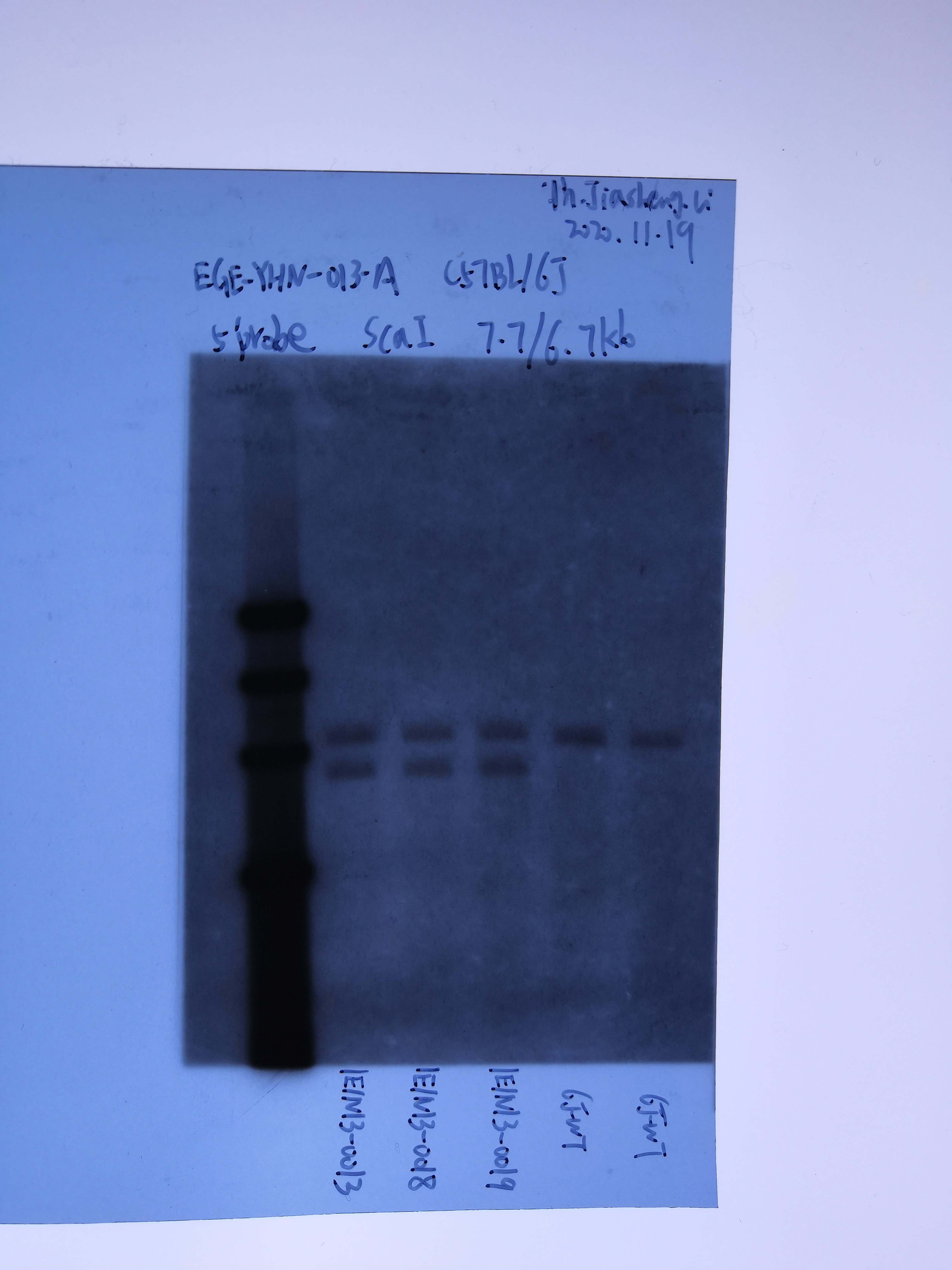

Supplement: Figure 1—source data 2. [file elife-93413-fig1-data2.zip › Figure1C_SourceData/Uncropp original EGE-YHN-013-A 5'probe ScaI heter-mice Southern blot original data-2.tif]

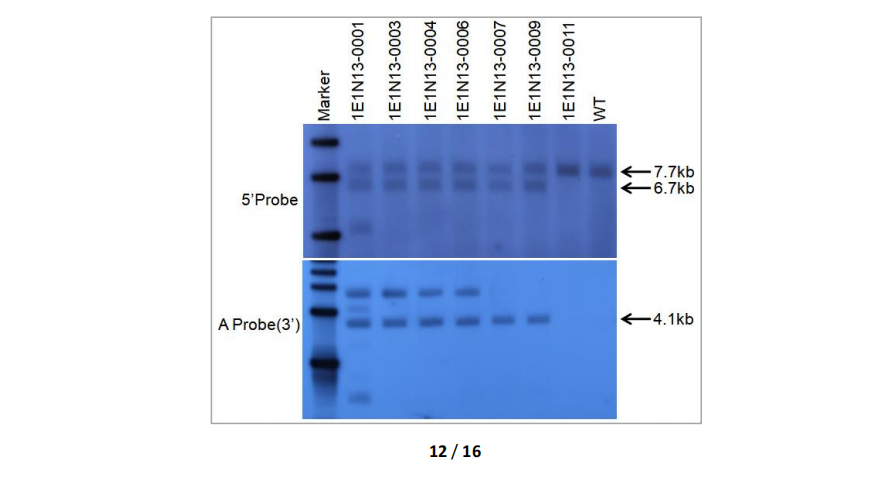

Supplement: Figure 1—source data 2. [file elife-93413-fig1-data2.zip › Figure1C_SourceData/labelled Original images for Figure 1-C-b1 .tif]

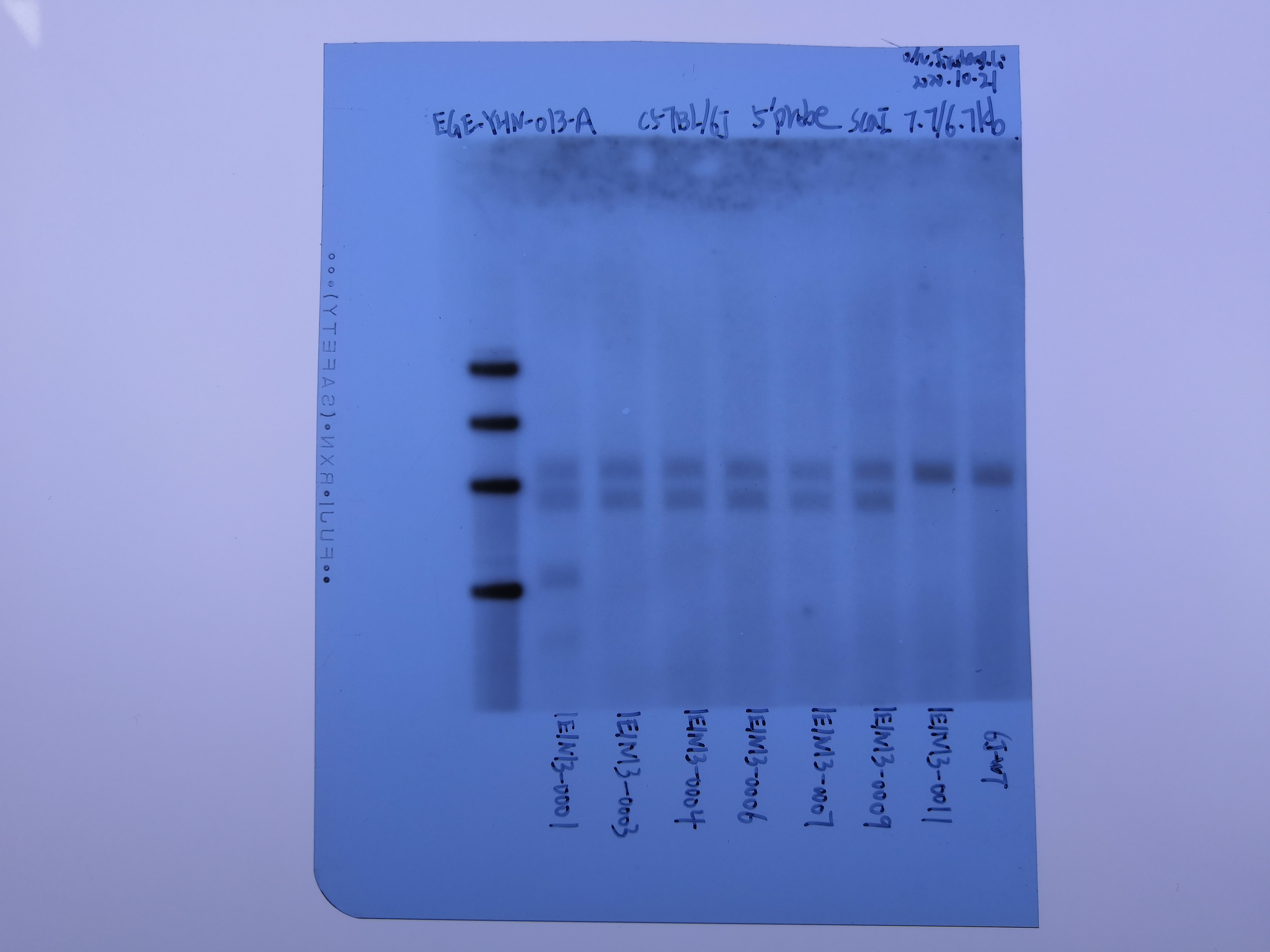

Supplement: Figure 1—source data 2. [file elife-93413-fig1-data2.zip › Figure1C_SourceData/Uncropp original , EGE-YHN-013-A 5'probe ScaI heter-mice Southern blot original data1.tif]

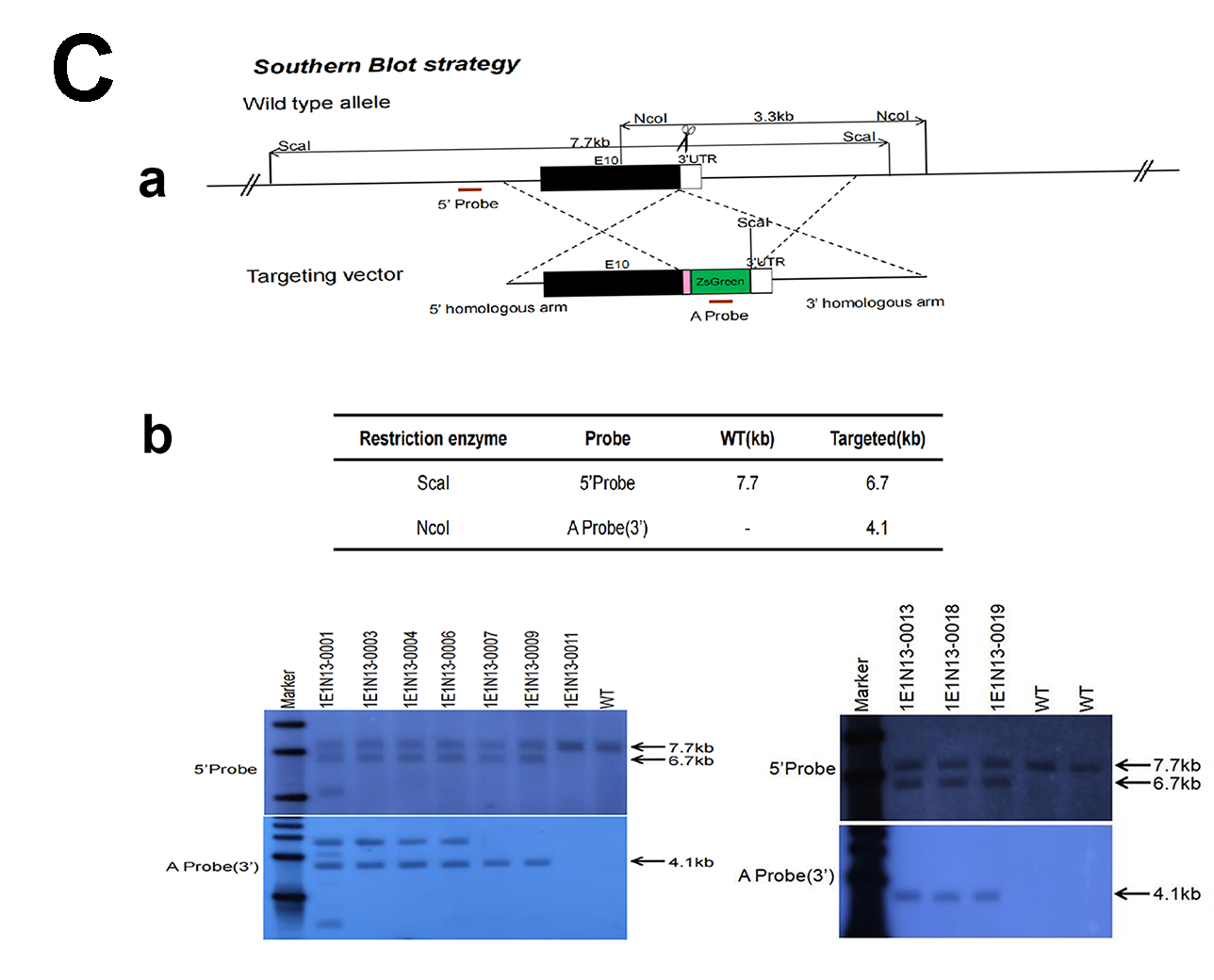

Supplement: Figure 1—source data 2. [file elife-93413-fig1-data2.zip › Figure1C_SourceData/labelled Fig. 1C.tif]
